# *Leishmania infantum* lipophosphoglycan induced-Prostaglandin E_2_ production in association with PPAR-γ expression via activation of Toll like receptors-1 and 2

**DOI:** 10.1038/s41598-017-14229-8

**Published:** 2017-10-30

**Authors:** Jonilson Berlink Lima, Théo Araújo-Santos, Milena Lázaro-Souza, Alan Brito Carneiro, Izabela Coimbra Ibraim, Flávio Henrique Jesus-Santos, Nívea Farias Luz, Sara de Moura Pontes, Petter Franco Entringer, Albert Descoteaux, Patrícia Torres Bozza, Rodrigo Pedro Soares, Valéria Matos Borges

**Affiliations:** 1 Gonçalo Moniz Institut, Oswaldo Cruz Foundation (FIOCRUZ-BA), 40296-710 Salvador, BA Brazil; 2 Center of Biological Sciences and Health, Federal University of Western Bahia (UFOB), 47808-021 Barreiras, BA Brazil; 3 Federal University of Bahia (UFBA), 40110-170 Salvador, BA Brazil; 4 Laboratory of Immunopharmacology, Oswaldo Cruz Institut, FIOCRUZ-RJ, 21040-900 Rio de Janeiro, RJ Brazil; 5 René Rachou Institut, Oswaldo Cruz Foundation (FIOCRUZ-MG), 30190-002 Belo Horizonte, MG Brazil; 6 Federal University of Rio de Janeiro (UFRJ), NUPEM, Campus Macaé, 27933-378 Macaé, RJ Brazil; 7 Institut National de la Recherche Scientifique, Institut Armand-Frappier, H7V 1B7 Laval, Canada

## Abstract

Lipophosphoglycan (LPG) is a key virulence factor expressed on the surfaces of *Leishmania* promastigotes. Although LPG is known to activate macrophages, the underlying mechanisms resulting in the production of prostaglandin E_2_ (PGE_2_) via signaling pathways remain unknown. Here, the inflammatory response arising from stimulation by *Leishmania infantum* LPG and/or its lipid and glycan motifs was evaluated with regard to PGE_2_ induction. Intact LPG, but not its glycan and lipid moieties, induced a range of proinflammatory responses, including PGE_2_ and nitric oxide (NO) release, increased lipid droplet formation, and iNOS and COX2 expression. LPG also induced ERK-1/2 and JNK phosphorylation in macrophages, in addition to the release of PGE_2_, MCP-1, IL-6, TNF-α and IL-12p70, but not IL-10. Pharmacological inhibition of ERK1/2 and PKC affected PGE_2_ and cytokine production. Moreover, treatment with rosiglitazone, an agonist of peroxisome proliferator-activated receptor gamma (PPAR-γ), also modulated the release of PGE_2_ and other proinflammatory mediators. Finally, we determined that LPG-induced PPAR-γ signaling occurred via TLR1/2. Taken together, these results reinforce the role played by *L*. *infantum-*derived LPG in the proinflammatory response seen in *Leishmania* infection.

## Introduction

Visceral leishmaniasis (VL) is caused by species from the *Leishmania donovani* complex. In the New World and Europe, this disease is mainly linked to *L*. *infantum*, which is widespread throughout Latin America, including Brazil, accounting for approximately 90% of all VL cases. VL is one of the most severe types of leishmaniasis and can prove lethal if untreated. Several determinants of VL virulence/pathogenicity have been attributed to intraspecies variation among *L*. *infantum* strains, in addition to host immune response^1^.

Lipophosphoglycan (LPG) is a major *Leishmania* surface glycoconjugate. This pleiotropic virulence factor is crucial during host-parasite interaction in both vertebrate and invertebrate hosts^2^. At early infection stages, LPG inhibits the complement system, favors opsonization by macrophages, impairs phagolysosome maturation and inhibits protein kinase C activation^3–9^. LPG is comprised of a glycan core, to which carbohydrate residues are added by different enzymes^10^. Biochemically, LPGs demonstrate intra- and inter-species variability with regard to repeat units. For example, *L*. *infantum* exhibits three types of LPG (I, II and III), each with different glucosylation levels, whereas *L*. *donovani* and *L*. *braziliensis* are devoid of side-chains in their repeat units^11–13^. While these biochemical differences are determinant to a pleiotropic range of immune responses in the vertebrate host, the isolated immunomodulatory properties of intact *L*. *infantum* LPG and/or its glycan and lipid moieties remain to be determined.

LPGs of different *Leishmania* species are potent agonists of Toll-like receptors (TLRs), especially TLR2 and TLR4^14–18^. Early studies revealed that purified LPG from *L*. *major* activates TLR2 and induces the nuclear translocation of NF-kB via Myd88^19,20^. Regarding New World species of *Leishmania*, purified LPG from *L*. *infantum* and *L*. *braziliensis* exhibits antagonic properties. LPG from *L*. *braziliensis* and *L*. *mexicana* are very pro-inflammatory, activating the ERK 1/2, JNK and p38 pathways via TLR2^18,21^. An interesting feature in the pathway activation profile of *L*. *infantum* LPG is its ability to gradually induce JNK and p38 subsequent to MAPK activation, whereas the profile associated with *L*. *brasiliensis* LPG is very transient^21^. *L*. *amazonensis* LPGs have been reported to induce NO, TNF-α and IL-6 via TLR4 and but were not capable of translocating NF-kB^17^.

The balance between lipid mediators, mainly the eicosanoids leukotriene B_4_ (LTB_4_) and prostaglandin E_2_ (PGE_2_), is also an important component of the inflammatory response to and outcome of infection by intracellular pathogens^22^. Previous *in vitro* studies have demonstrated the role of LTB_4_ as a parasite killing mechanism, while PGE_2_ favors *Leishmania* survival^23–28^. More recently, lipid mediators have been identified as biomarkers during cutaneous^29,30^ and visceral leishmaniasis^31^. In addition, *L*. *amazonensis* or its lipophosphoglycan is known to induce neutrophil activation, degranulation and leukotriene B_4_ (LTB_4_) production^23^. Nevertheless, the role of LPGs in the macrophage activation of eicosanoid production pathways, such as PGE_2,_ is still unknown.

It has been well established that during inflammatory responses, intracellular organelles known as lipid droplets (LDs) are the main sites where enhanced eicosanoid production takes place, e.g. PGE_2_ production by cyclooxygenase-2 (COX-2)^32^. The ability of *L*. *infantum*-infected cells to induce increased LD formation was recently demonstrated^33^. PGE_2_ production machinery has been associated with PPAR-γ expression. The COX-2 gene promoter presents a PPAR-γ response element (PPRE), which indicates that the induction of PPARγ regulates COX-2 expression and, consequently, PGE_2_ production^34^. In addition, PPAR-γ also participates in TLR2-induced LD formation and PGE_2_ production in macrophages^35^. In this scenario, COX-2 plays an important role as a downstream pathway that is engaged during TLR activation by *Leishmania* parasites or their PAMPs^36^.

In the present study, we evaluate the role of *L*. *infantum* LPG and its derived fragments in triggering a proinflammatory immune response related with PGE_2_ production by macrophages. Only intact LPG, not its derived moieties, was found to trigger the TLR-1/2-induced signaling pathway via PPAR-γ activation, thereby contributing to a PGE_2_-associated inflammatory response.

## Results

Intact *Leishmania infantum* LPG stimulates macrophage activation in a proinflammatory response associated with PGE_2_ production.

**Figure 1:**
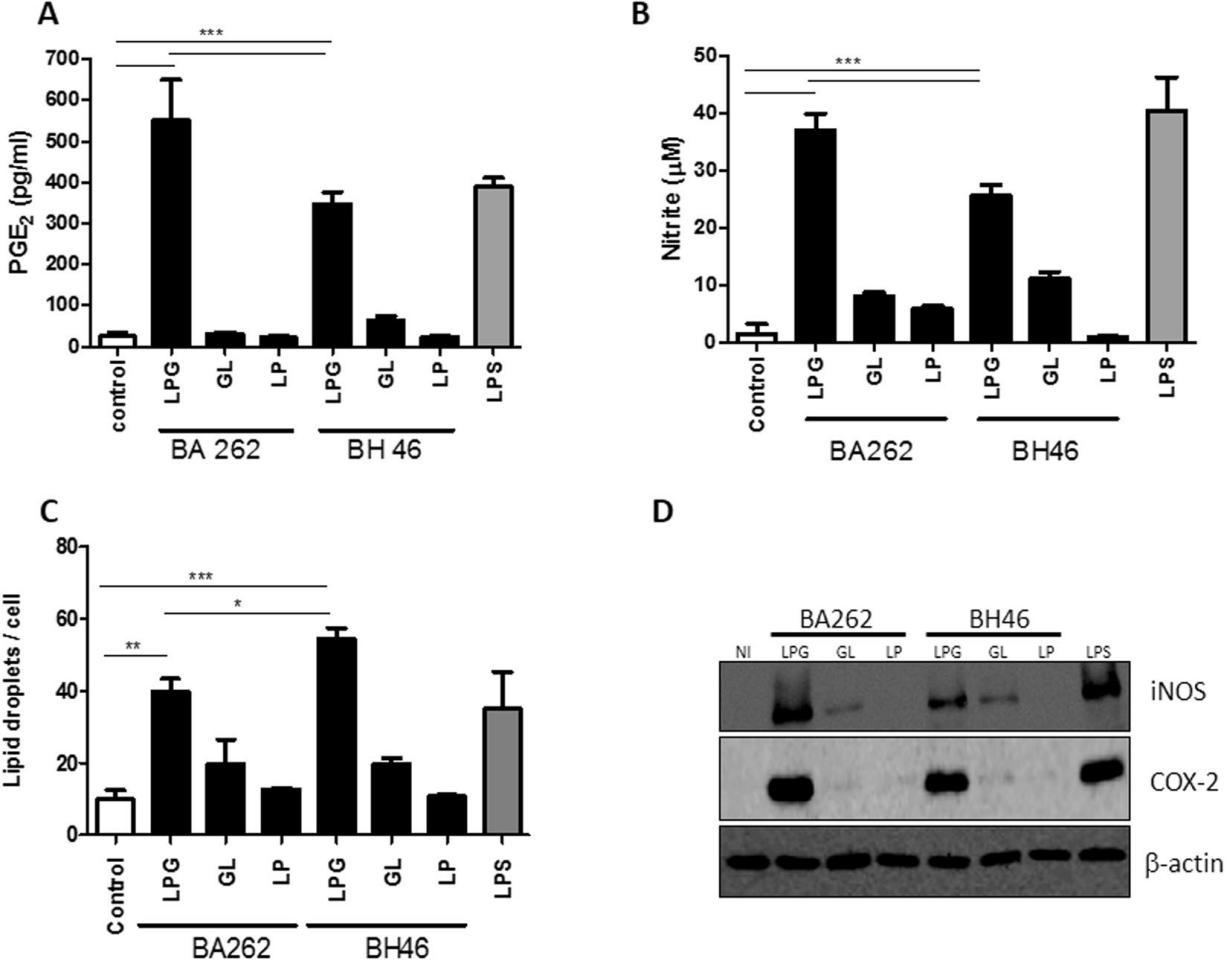
Intact LPG extracts induce a prostaglandin E2-associated inflammatory response. Bone Marrow Derived Macrophages (BMDM) primed with IFN-γ (100ng/mL) were stimulated for 24 h with 10 µg/mL of intact LPG extract or LPG-derived fragments: lipids (LP) or glycans (GL) from the BA262 or BH46 strains. LPS (500 ng/ml) was used as a positive control. PGE_2_ (**A**) and NO (**B**) were measured in culture supernatants. (**C**) Lipid droplets were enumerated on slides stained with osmium tetroxide. Bars represent means ± SD of three representative experiments performed in triplicate. ANOVA test followed by Student Newman-Keuls post-test was used to multiple comparison among experimental groups (*p < 0.05, **p < 0.01 and ***p < 0.001). (**D**) iNOS and COX-2 induction were evaluated by Western Blot 24 h after LPG stimulation.

Differences have been described with regard to intact *L*. *infantum* LPG and its lipid and glycan moieties derived from BA262 and BH46 strains in terms of the biochemical structure of glycidic side chains^13^ (Supplemental Fig. 1). Here, were tested whether these distinct LPG molecules are capable of inducing differential macrophage activation *in vitro*. IFN-γ-primed bone marrow-derived macrophages (BMDM) were incubated with LPG from BA262 or BH46 strains for 24 hours. Intact LPG from the BA262 and BH46 strains, but not their lipid and glycan moieties, induced PGE_2_ and NO production (Fig. 1A and B). Similarly, an increased number of LDs was observed only in intact LPG-stimulated cells (Fig. 1C). To demonstrate the involvement of LPG in the activation of enzymes implicated in PGE_2_ and NO production, protein expression of COX-2 and iNOS was determined by Western Blotting. IFN-γ-primed BMDMs stimulated for 24 hours with intact LPG, lipid and glycan moieties, exhibited robust iNOS and COX-2 expression only under stimulation by intact LPG (Fig. 1D).

LPG from the BA262 strain was chosen for the further experiments in order to better represent the most common unbranched LPG in *L*. *infantum*, since this molecule was well characterized as a type I LPG, found in the majority of strains (90%), including those from Brazil, Africa and Europe. On the other hand, BH46 strain (type III LPG) is only found in 5% of the strains^13^.

### COX-2 is involved in *L*. *infantum* LPG-induced PGE_2_ production

**Figure 2:**
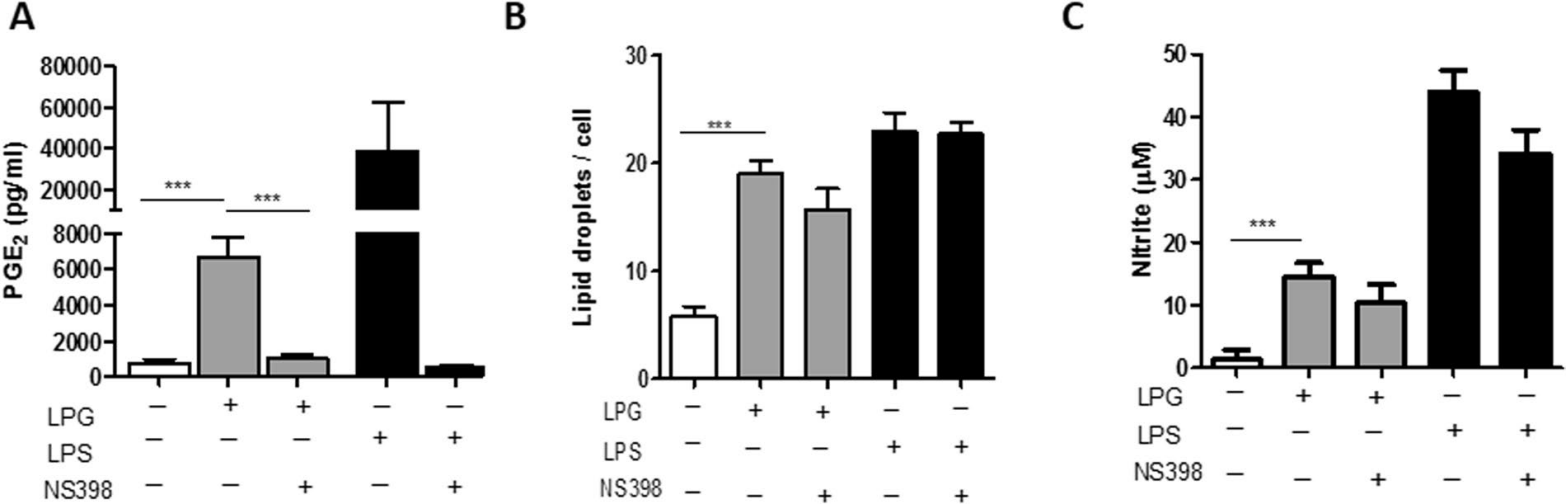
NS398 inhibits *L*. *infantum* LPG-induced PGE2 production, but did not alter lipids droplet formation or nitric oxide production. IFN-γ-primed BMDM were preincubated for 1 h with NS-398 (1μM), a specific COX-2 inhibitor, then stimulated for 24 h with 10 µg/mL purified intact LPG extract from the BA262 *L*. *infantum* strain or LPS (500 ng/ml) as a positive control. Graphs depict (**A**) PGE_2_ release, (**B**) lipid droplet quantification and (**C**) nitric oxide production. Bars represent means ± SD of two representative experiments performed in quadruplicate. ANOVA test followed by Student Newman-Keuls post-test was used to multiple comparison among experimental groups (***p < 0.001).

Since intact *L*. *infantum* LPG from the BA262 strain was capable of inducing PGE_2_ production in IFN-γ BMDM (Fig. 1A–D), COX-2 inhibition was performed by pretreatment of cells with NS-398, a selective COX-2 inhibitor, which blocked LPG-induced PGE_2_ production (Fig. 2A). In contrast, LDs formation (Fig. 2B) and NO production (Fig. 2C) were shown to be unaffected by NS-398 treatment, suggesting that COX-2 activity is not required for LD formation or NO production.

### Intact *L*. *infantum* BA262-derived LPG induces MAPK phosphorylation and a proinflammatory response

**Figure 3:**
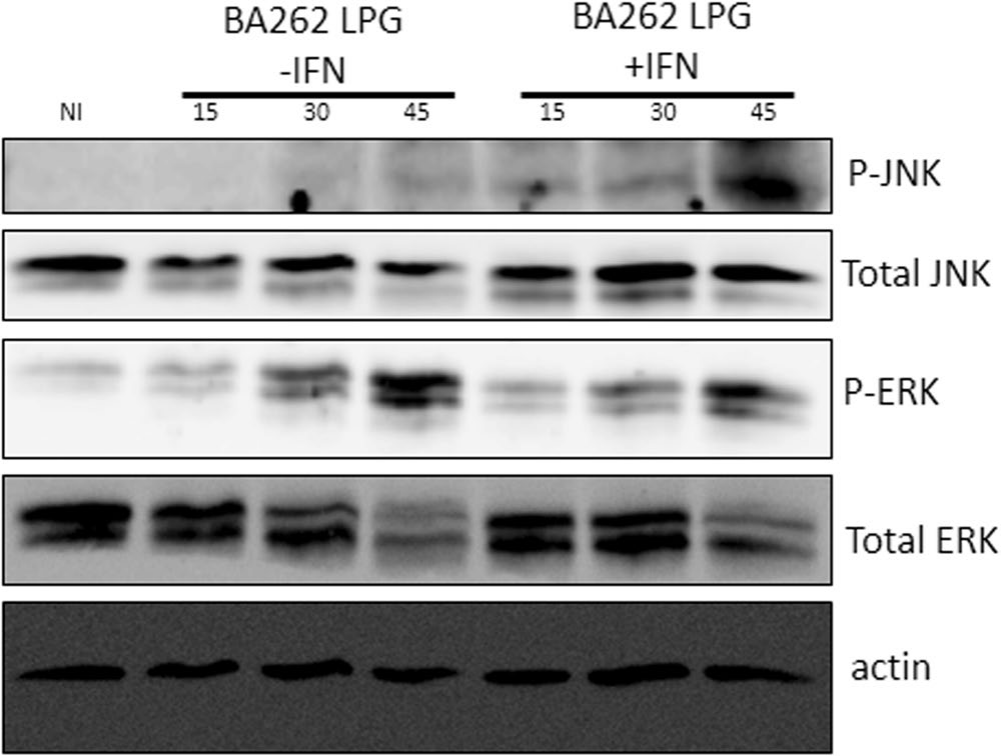
*L*. *infantum* LPG induces rapid ERK1/2 and JNK phosphorylation. BMDM were primed or not with IFN-γ (100 ng/mL) and stimulated with 10 µg/mL of purified intact LPG extract from the BA262 strain for 15, 30 and 45 min. Bands indicate ERK1/2 and JNK phosphorylation in cell lysates evaluated by Western Blot.

**Figure 4:**
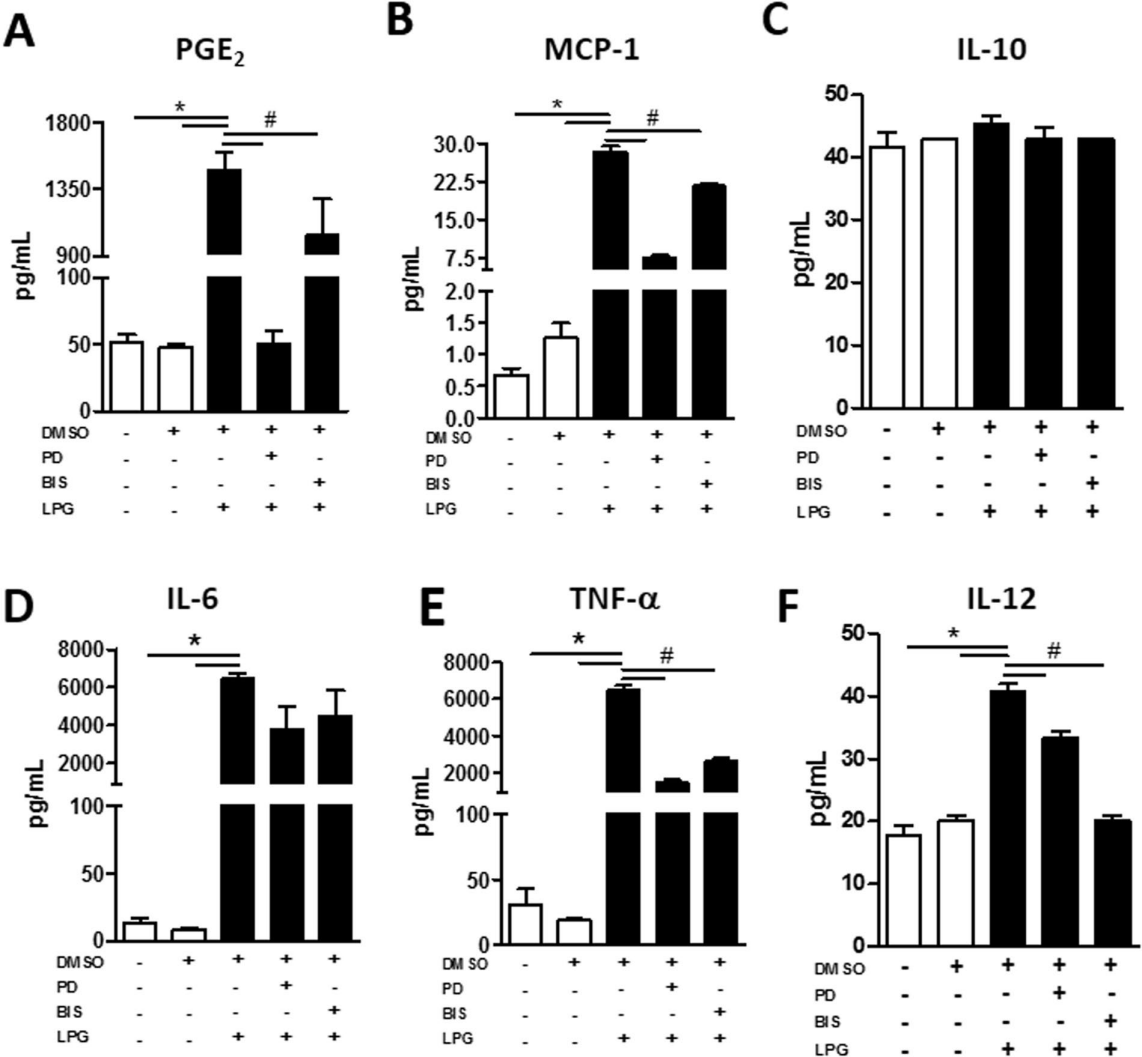
ERK-1/2 and PKC signaling induced by BA262 *L*. *infantum* LPG drives PGE2 and proinflammatory mediator production. Murine macrophages were pretreated for 1 h with 50 µM PD98059 (ERK-1/2 inhibitor) or 20 μM BIS I (PKC inhibitor) and stimulated for 24 h with 10 µg/mL purified intact LPG extract from the BA262 strain. (**A**) PGE_2_, (**B**) MCP-1, (**C**) IL-10, (**D**) IL-6, (**E**) TNF-α, (**F**) IL-12p70 levels were measured in culture supernatant. Bars represent means ± SD of two representative experiments in quintuplicate. ANOVA test followed by Student Newman-Keuls post-test was used to multiple comparison among experimental groups (*p < 0.05 compared to the control group; ^#^p < 0.05 compared to other groups).

*Leishmania* sp. LPG has been shown to rapidly induce MAP kinase activation^18,21^, which was confirmed herein by ERK and JNK activation via Western blotting of BMDMs stimulated with intact *L*. *infantum* LPG (Fig. 3). We then verified if the inhibition of ERK1/2 (by PD98059) and PKC (by BIS I) would influence the release of LPG-induced proinflammatory mediators. LPG stimulation alone was shown to induce PGE_2_, MCP-1, IL-6, TNF-α and IL-12p70 production (Fig. 4), but did not affect levels of IL-10. When ERK phosphorylation was inhibited, reduced LPG induced-production of PGE_2_, MCP-1, TNF-α and IL-12p70 was observed. Furthermore, PKC inhibition reduced TNF-α and IL-12p70 production. Interestingly, the inhibition of both signaling pathways resulted in decreased IL-6 production in the presence of LPG, yet IL-10 was unaffected (Fig. 4).

### PPAR-γ regulates TLR1/2 activation by LPG stimulation

**Figure 5:**
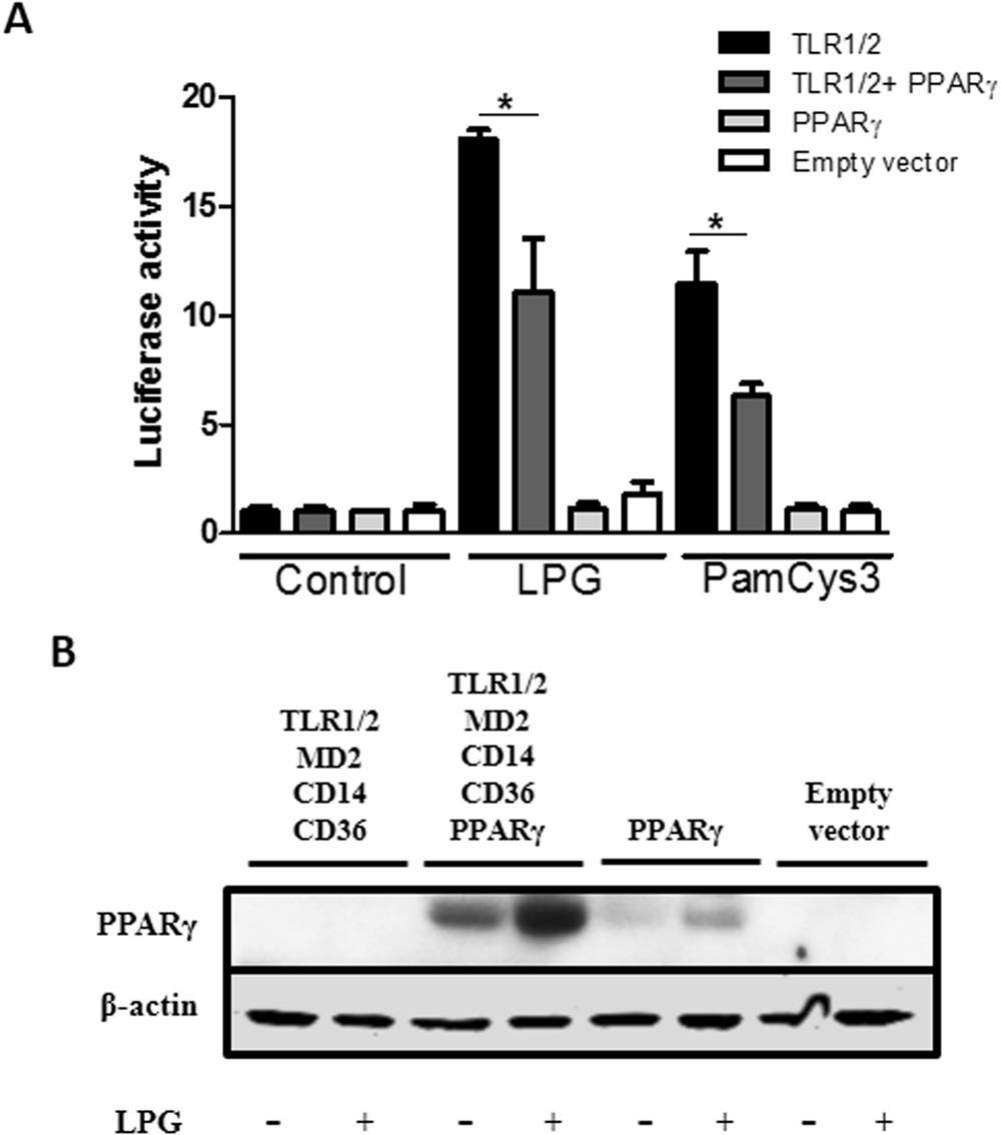
TLR-1/2-induced PPAR-γ signaling is trigged by BA262 *L*. *infantum* LPG (**A**) HEK 293 T cells were transfected with four different constructions: (i) TLR-1/2, MD2, CD14 and CD36; (ii) TLR-1/2, MD2, CD14, CD36 and PPAR-γ; (iii) PPAR-γ transfection control; (iv) empty vector. After 24 h of transfection, cells were stimulated with 10 µg/mL of purified intact LPG extract from the BA262 strain, 1 nM of Pam3CSK4 or medium alone (control). After 4 h, transfected HEK 293T cells were lysed and luciferase activity was assessed by NF-κB activation using a Dual-Luciferase Reporter Assay System. Bars represent means ± SD of two representative experiments performed in triplicate. ANOVA test followed by Tukeys’ post-test was used to multiple comparison among experimental groups (*p < 0.5). (**B**) PPAR-γ expression was evaluated by western blotting after LPG stimulation in the four groups of transfected HEK 293 T cells using β-actin as a housekeeping gene.

**Figure 6:**
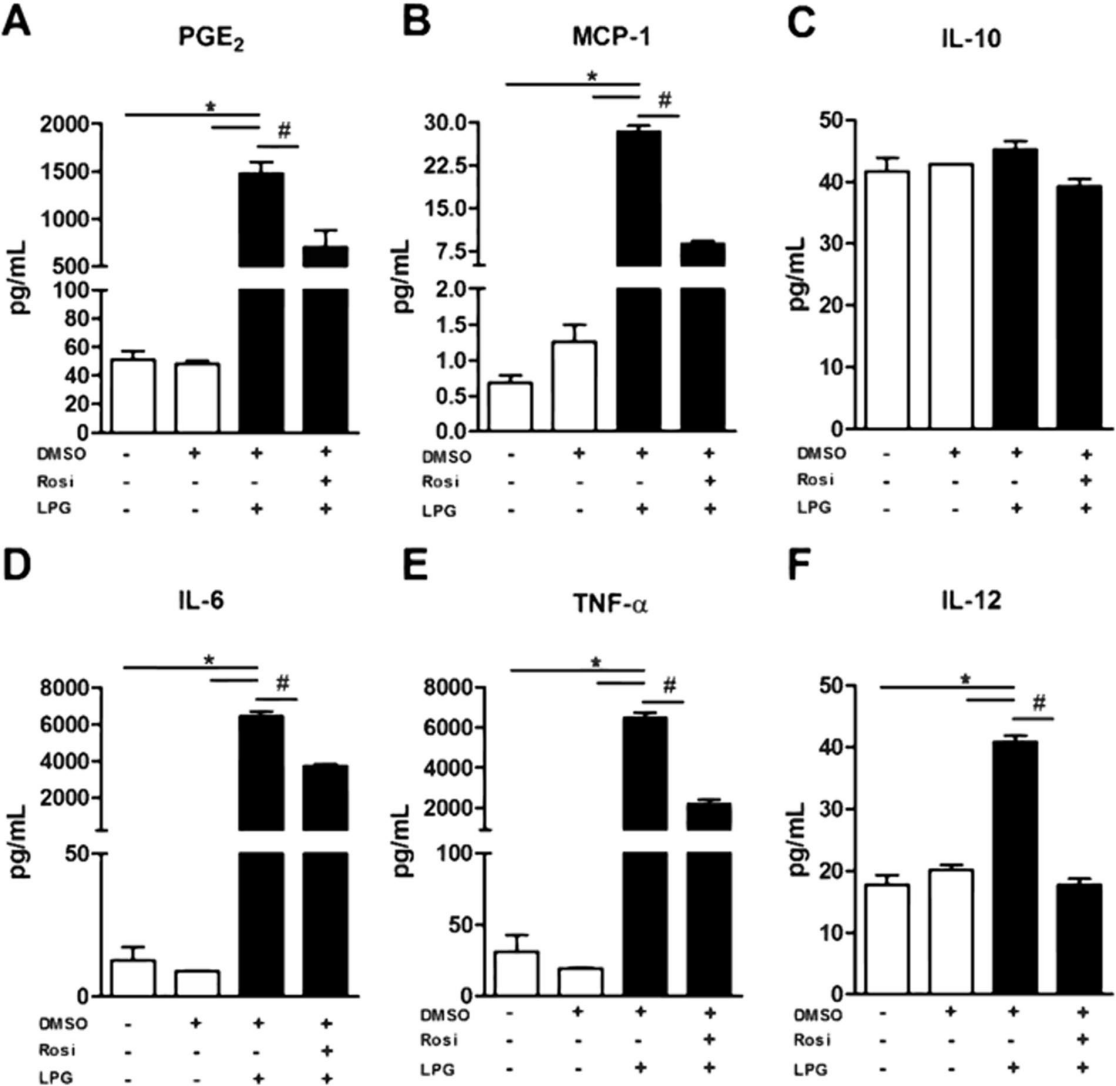
PPAR-γ signaling induced by BA262 *L*. *infantum* LPG drives PGE2 and pro-inflammatory mediator production. Murine macrophages were treated for 24 h with 10 µM rosiglitazone, a PPAR-γ agonist (10 µM), then stimulated for 24 h with 10 µg/mL of purified intact LPG extract from the BA262 strain. Levels of (**A**) PGE2, (**B**) MCP-1, (**C**) IL-10, (**D**) IL-6, (**E**) TNF-α, (**F**) IL-12p70 were measured in culture supernatant. Bars represent means ± SD of two representative experiments in quintuplicate. ANOVA test followed by Student Newman-Keuls post-test was used to multiple comparison among experimental groups (*p < 0.05 compared to the control group; ^#^p < 0.05 compared to other groups).

Since LPG has been shown to activate TLR-1/2^21,28,37^, we employed a luciferase reporter assay using HEK293 cells transfected with different combinations of TLR-1 and -2, including PPAR-γ to confirm the downstream response. TLR1 and TLR2 were found to be activated in the presence of *L*. *infantum* LPG and PamCys3 (a TLR-1/2 agonist, positive control) (Fig. 5A). TLR1/2 activation was able to induce PPAR-γ expression (Fig. 5B). These findings indicate that TLR1/2 recognize *L*. *infantum* LPG with further downstream regulation by PPAR-γ. Next, to investigate the potential role of PPAR-γ in the regulation of an LPG-induced inflammatory response in activated macrophages, cells were incubated in the presence or absence of rosiglitazone, a PPAR-γ agonist, prior to the addition of purified LPG (Fig. 6). PPAR-γ activation decreased the levels of PGE_2_, MCP-1, IL-6, TNF-α and IL12p70, whereas IL-10 production remained unaffected (Fig. 6). These data reinforce the role of PPAR-γ activation in the downregulation of an LPG-induced inflammatory response.

## Discussion

Glycoconjugates are important PAMPs in trypanosomatids that play pivotal roles during parasite-host interaction. In this context, LPG is a key virulence factor expressed at the surface of *Leishmania* promastigotes. Many functions have been attributed to this molecule in both vertebrate and invertebrate hosts^15,16,38,39^. While the roles of LPG in the innate immune response have been well established, the literature contains scarce data regarding the involvement of LPGs in lipid mediator production. Previous studies by our group have shown that *L*. *amazonensis*-derived LPG induced LTB_4_ production by human neutrophils^23^. Here we investigated whether the LPGs and/or LPG-derived moieties from different *L*. *infantum* strains were capable of triggering PGE_2_ and proinflammatory cytokine production.

While comprehensive knowledge surrounding the critical aspects of LPG structure and functioning remains limited, one distinguishing feature of *L*. *infantum-*derived LPG is that it exhibits intraspecies polymorphisms (type I, II and III). Since the number of β-glucose residues branching off the repeat units is critical to NO production^13^, we purified LPGs from *L*. *infantum* strains BA262 (type I) and BH46 (type III), as well as their lipid and glycan motifs. Consistent, only intact LPG from both *L*. *infantum* strains was shown to trigger robust nitrite production and iNOS expression by murine macrophages.

Glycoconjugates are GPI-anchored structures that may be released in the extracellular milieu either due to the action of different phospholipases, or as surface components of extracellular vesicles^40–42^. These include LPGs, GIPLs, proteophosphoglycans (PPGs), phosphoglycans (PGs), secreted acid phosphatases (*s*AP) and glycoprotein 63 (GP63)^43^. In the case of *L*. *major* LPG and *Trypanosoma cruzi* glycosylphosphatidylinositol (GPI)-mucins, disruption of the lipid anchor was shown to abrogate proinflammatory activity and NO production^19,44^. In addition, treatment of *L*. *braziliensis* GIPLs with phosphatidylinositol-specific phospholipase C (PI-PLC) was shown to restore NO production by murine macrophages^16^. In the other hand, here the treatment of LPG from both *L*. *infantum* strains with phosphatidylinositol-specific phospholipase C (PI-PLC) decreased levels of NO, PGE_2_, lipid body formation and COX-2 expression by macrophages. By contrast, the diminished action evidenced by the purified lipid and glycan moieties as compared to that of intact LPG, suggests that these fragments may act synergistically. Another possibility could be that the lipid anchor is required for the insertion of the highly-charged phosphoglycan moiety into the membrane.

Although both intact LPG from BA262 and BH46 induced a proinflammatory response, PGE and NO induction were higher in the supernatant of macrophages stimulated with BA262 LPG, while droplet formation increased in cells stimulated with BH46 LPG. This finding may be explained by the fact that lipid droplets serve as reservoirs that provide substrate for the enzymatic activity of COX-2 (which produces PGE_2_), as well as 5-lipoxigenase (5-LO, which produces leukotrienes and lipoxins)^45^. In addition, the utilization of lipid droplet-derived arachidonic acid by both COX-2 or 5-LO is not well understood. Accordingly, it is possible that discrepancies between lipid droplet quantification and PGE_2_ measurements results from the activation of enzymatic pathways other than COX-2. Interestingly, treatment with NS-398, an irreversible COX-2 inhibitor, was shown to reduced PGE_2_ levels, yet LD formation and NO production remained unaffected, which suggests that the enhanced LD formation induced by BH46 LPG is unrelated to NO or PGE2 production. This reinforces the notion that LPG integrity is functionally required for the immunomodulation of host cells.

A diverse range of PAMPs and DAMPs can activate ERK-1/2 and JNK signaling pathways^46–48^. Early studies using synthetic *Leishmania* PGs demonstrated the effect of this glycoconjugate in subverting IL-12 production via the ERK pathway^49^. Later, LPG-induced MAPKs were investigated in other New World *Leishmania* species^17,21^. The ability of LPGs to induce MAPKs is variable, and is perhaps species-specific. For example, LPGs derived from *L*. *mexicana*, *L*. *infantum* (BH46 strain), *L*. *enriettii*, *L*. *braziliensis* and *L*. *amazonensis* were shown to differentially activate ERK-1/2, p38 and JNK pathways^17,18,21,37^. On the other hand, GIPLs from *L*. *infantum* and *L*. *braziliensis* are potent inhibitory molecules that do not activate ERK, p38 and JNK^16^. Here, LPG from *L*. *infantum* BA262 was shown to induce ERK-1/2 and JNK phosphorylation in macrophages, which confirms that the mechanisms underlying MAPKs pathways participate in signaling following stimulation with glycoconjugates. Indeed, the pharmacologic inhibition of PKC and ERK-1/2 signaling was found to affect the production of some cytokines and PGE_2_ under stimulation with intact LPG.

Similarly to MAPKs, the secretion of proinflammatory cytokines and chemokines varies greatly among different *Leishmania* species, not only in the Old World, but also in species found in the New World. Inflammatory signaling and cytokine production are essential to parasite control. During *Leishmania* infection, a proinflammatory response that results in tissue damage could be triggered by LPG or other molecules secreted by the parasite^50–53^. Thus, the mechanisms underlying immune response activation by *Leishmania* PAMPs is crucial in determining the inflammatory balance that defines whether an infection will be controlled or exacerbated^54^. Interestingly, following LPG stimulation, dermotropic species exhibit a more exacerbated proinflammatory profile, whereas viscerotropic species present immunosuppression. This is the case with *L*. *infantum* LPG which was not able to translocate NF-kB (strain BH46), and induced lower levels of NO and cytokines (TNF-α, IL-1β and IL-6) compared to *L*. *braziliensis*,^21^. Here, LPG from strain BA262 successfully induced cytokine and chemokine production by murine macrophages.

It is interesting to note that a COX-2 antagonist was not capable of reducing LD formation and NO production, which indicates that other pathways may be involved in LPG-induced PGE_2_ production. PPAR-γ signaling has been shown to coordinate the inflammatory immune response, as well as COX-2 activation and the induction of lipid mediators in a variety disease models^36,55–61^. The activation of PPAR-*γ* by intracellular mycobacterial infection induces lipid droplet formation and PGE_2_ production, as well as favoring mycobacterial survival through the downmodulation of NF-κB signaling^35,62^. Similarly, *L*. *donovani* infection induces PPAR-*γ* activation and promotes parasite survival, whereas the inhibition of PPAR-*γ* facilitates *Leishmania* clearance^63^. By contrast, PPAR-γ activation in *L*. *mexicana*-infected macrophages was shown to control parasite burden by oxidative stress, while selectively regulating prostaglandin production, yet with no reduction in PGE_2_ production following infection^36^. On the other hand, *L*. *infantum* infection or stimulation by LPG induced the production of heme oxygenase 1 (HO-1) in murine macrophages in a manner not dependent on PPAR-γ^64^. The present results using a HEK luciferase reporter systems evidence that PPAR-γ is induced by *L*. *infantum* intact LPG. In addition, an LPG-induced inflammatory response was successfully reverted by PPAR-γ activation. Rosiglitazone, a PPAR-γ agonist was shown to reduce the production of PGE_2_ and MCP-1, in addition to a range of pro-inflammatory cytokines, suggesting the involvement of a mechanism other than NF-κB signaling. Although the significance of PPAR-γ activation by *L*. *infantum* LPG is not fully understood, this induction could represent a strategy for the control of an LPG-mediated inflammatory response that is capable of inducing tissue damage during initial infection stages.

**Figure 7:**
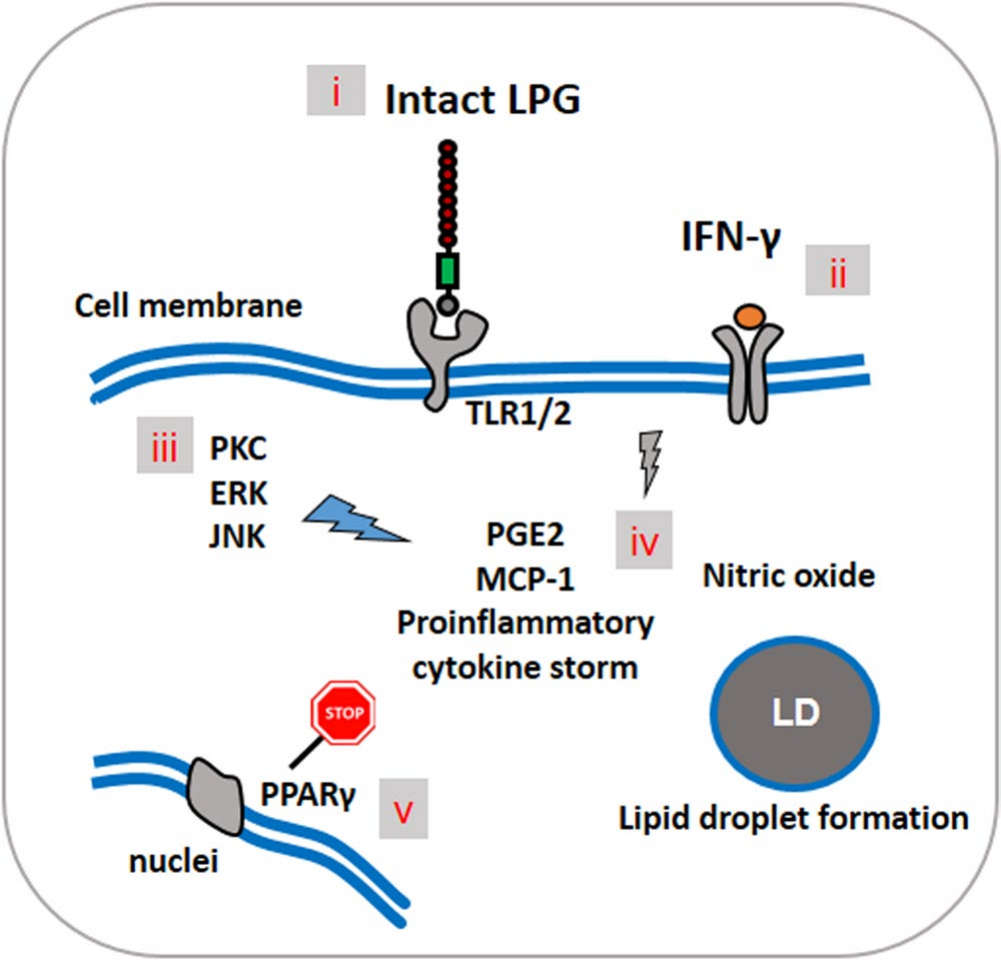
Illustration of *L*. *infantum* LPG-induced PGE_2_-associated inflammatory response. Intact LPG is recognized by TLR-1/2 (i) on the membranes of IFN-γ−primed macrophages (ii), triggering the MAPs kinase signaling pathway, leading to the phosphorylation of PKC, ERK and JNK (iii). This process induce thes PGE_2_ production and lipid droplet formation, as well as NO and MCP-1 production in addition to a proinflammatory cytokine storm (iv). PPARγ regulates the LPG-induced PGE_2_-associated inflammatory response in a feedback mechanism (v). Illustration by Théo Araújo-Santos.

Based on the present results, we have developed an illustrative model (Fig. 7) to explain the involvement of TLR1/2 in the recognition of *L*. *infantum* LPG. A previous investigation revealed that while TLR2 participates in the induction of PGE_2_ by *Leishmania*, the identity of the ligand(s) involved in this activation remains undetermined^28^. Using a HEK293 reporter cell system, we have demonstrated that intact LPG is capable of activating TLR-1/2 consequently inducing the expression of PPAR-γ. Finally, the TLR-1/2 activation by LPG engages kinase-signaling proteins, such as PKC, ERK1–2 and JNK, that contribute to a PGE_2_-associated inflammatory response. In parallel, as PPAR-γ partially regulates inflammatory activation, this reinforces the notion that PPAR-γ is a critical regulator of LPG-induced inflammatory signaling. Further study is needed to more comprehensively address the role played by LPG in the outcome of *L*. *infantum* infection.

## Methods

### Antibodies and Reagents

RPMI 1640 medium and L-glutamine, penicillin, and streptomycin were obtained from Invitrogen (Carlsbad, CA, USA). Nutridoma-SP was purchased from Roche (Indianapolis, In, USA). NS-398, Rosiglitazone and the PGE_2_ enzyme-linked immunoassay (EIA) kit were acquired from Cayman Chemical (Ann Arbor, MI, USA). Osmium tetroxide (OsO_4_) was obtained from Electron Microscopy Science (Fort Washington, PA, USA). AquaPolymount was purchased from Polysciences (Warrington, PA, USA). PD 98059, 29-Amino-39-methoxyflavone and Bisindolylmaleimide-I, 2-[1-(3-Dimethylaminopropyl)-1H-indol-3-yl]-3-(1H-indol-3-yl)-maleimide (BIS) were obtained from Merck-Calbiochem (Darmstadt, Hessen, Germany). S-[2,3-bis(palmitoyloxy)-(2-RS)-propyl]-N-palmitoyl-(R)-Cys-Ser-Lys4-OH (PamCys3), a TLR2/TLR1 agonist, was acquired from Invitrogen (San Diego, CA, USA) and a Bead Array mouse inflammation kit was purchased from BD Biosciences (San Jose, CA, USA). Primary β-actin, pERK, ERK, P-JNK and JNK antibodies were obtained from Cell Signaling (Danvers, MA, USA), while Cox-2 and iNOS antibodies were purchased from Calbiochem (San Diego, CA, USA).The Dual-Luciferase Reporter Assay System was acquired from Promega (Madison, WI, USA).

### Ethics Statement

All experiments were performed in strict accordance with the recommendations of the Brazilian Council for the Control of Animal Experimentation (CONCEA). The Oswaldo Cruz Foundation Institutional Review Board for Animal Experimentation (CEUA-IGM-FIOCRUZ) approved all present experimental protocols (Protocol number: 021/2015).

### Animals

Inbred male C57BL/6 mice aged 6–8 weeks were obtained from the animal care facility at the Gonçalo Moniz Institute, Oswaldo Cruz Foundation (IGM-FIOCRUZ, Bahia-Brazil). The animals were kept at a temperature of 24 °C with free access to food and water under regular 12-hour cycles of light and darkness.

### *Leishmania infantum* culture and LPG Extraction

*L*. *infantum* (strains MCAN/BR/89/BA262 and MHOM/BR/1970/BH46) promastigotes were cultured in M199 medium supplemented with 10% inactive FBS, 2 mM L-glutamine, 100 U/ml penicillin, 50 μg/ml streptomycin, 12.5 mM glutamine, 0.1 M adenine, 0.0005% hemin and 40 mM Hepes, adjusted to a pH 7.4, at 26 °C until late log-phase. LPG was extracted and purified as previously described^65^. LPG purity was checked using Chinese hamster ovary (CHO) cells transfected with TLR2 or TLR4, as reported elsewhere^17^. Purified LPG extract was treated with Phosphatidylinositol-specific phospholipase C (PI-PLC) to release the lipid and glycan moieties. Glycans and lipids were separated as previously described^66^.

### Macrophage culturing and IFN-γ activation

Bone Marrow Derived Macrophages (BMDM) were obtained from C57BL/6 mice. Cells were collected from femurs and differentiated in RPMI 1640, 20% FBS, 30% L-929 cell-conditioned media (LCCM), 2mM L-glutamine and 100 U/mL penicillin/Streptomycin at 36 °C under 5% CO_2_. BMDMs were collected after seven days and seeded on tissue culture plates in RPMI 1640 media, 10% FBS, 5% LCCM and 2 M L-glutamine. For cytokine quantification, peritoneal macrophages were obtained four days after intraperitoneal injection of 1 mL 3% thioglycolate solution in C57BL/6 mice. Peritoneal cells were harvested using 10 mL RPMI 1640 and then centrifuged at 400xg for 10 minutes. Macrophages (3 × 10^5^/well) were cultured in 1 mL of RPMI 1640 medium supplemented with 2mM L-glutamine, 100 U/mL penicillin and 100 µg/mL streptomycin. For activation, all macrophage cultures were stimulated with 100 U/mL recombinant IFN-γ for 24 hours. IFN-γ-primed macrophages were used in experimentation as unprimed cultures stimulated with LPG fail to induce NO^13^.

### PGE_**2**_, NO and lipid droplet quantification

PGE_2_ and NO was evaluated in the culture supernatants of IFN-γ-primed BMDM treated for 24 h with 10 µg/mL intact LPG extract or LPG-derived fragments (lipids or glycans) from the BA262 or BH46 strains. LPS (500 ng/ml) was used as a positive control. Levels of PGE_2_ were measured by EIA in the supernatant in accordance with manufacturer protocols. Nitrite concentrations were determined by Griess reaction. Lipid droplet quantification was performed in adhered cells fixed in 3.7% formaldehyde, then stained with osmium tetroxide as described previously^67^. Lipid droplets were counted by light microscopy using a 100x objective lens in 50 consecutively scanned macrophages. For the COX-2 enzyme inhibition assays, IFN-γ-primed BMDM were preincubated for 1 h with NS-398 (1 μM), a specific COX-2 inhibitor. Next, cells were stimulated with 10 µg/mL purified intact LPG extract only from the BA262 *L*. *infantum* strain, or LPS (500 ng/ml) as a positive control. After 24 h, PGE_2_, NO and lipid droplet quantification was performed as described above.

### Signaling pathway inhibition

For the inhibition assays, IFN-γ-primed peritoneal macrophages were preincubated for 1 h with: i) 20 μM BIS, an inhibitor of PKC; ii) 50 µM PD98059, an inhibitor of ERK-1/2; iii) 10 µM Roziglitazone, an agonist of PPAR-γ. Next, cells were stimulated for 24 h with purified intact 10 µg/mL LPG extract from only the BA262 *L*. *infantum* strain. PGE_2_ levels were measured in the supernatant as described above. Cytokines (TNF-α, IL-6 MCP-1, IL-12p70 and IL-10) were quantified using a Cytometric Bead Array (CBA) inflammatory kit mouse following manufacturer’s instructions.

### Western blot analysis

To evaluate the inflammatory response triggered by LPG stimulation, COX-2 and iNOS expression were determined by western blotting. IFN-γ-primed BMDM were stimulated with purified intact LPG extract or LPG-derived fragments from BA262 and BH46 *L*. *infantum* strains for 24 h. BMDMs were then lysed in RIPA buffer supplemented with a cocktail of protease and phosphatase inhibitors. Protein extracts were resolved by SDS-PAGE and transferred to a nitrocellulose membrane, then probed with Cox-2 and iNOS. Levels of phosphorylated MAPKs signaling pathways triggered by LPG stimulation were also examined by western blotting. BMDMs, primed or not with IFN-γ, were stimulated with 10 µg/mL purified intact LPG extract only from the BA262 strain for 15, 30 or 45 min., then probed with β-actin, pERK, ERK, P-JNK and JNK. Bands were detected using an Image Quant LAS 4010 (GE healthcare).

### HEK 293T cell transfection assay

For the transfection assays, HEK 293 T cells were plated in 12-well plates (5 × 10^5^ cells/well) after 24 h using Lipofectamine 2000 and Opti-MEM (Invitrogen) in accordance with manufacturer instructions. Four different groups were transfected: (1) TLR2, TLR1, MD2, CD14 and CD36; (2) TLR2, TLR1, MD2, CD14, CD36 and PPARγ; (3) PPARγ transfection control; (4) empty vector. For transfection, the following targets were added to the wells: 0.2 μg mouse TLR2, 0.8 μg mouse TLR1, 0.22 μg mouse MD-2, CD14 and CD36, 0.2 μg firefly luciferase reporter construct driven by an NF-κB-dependent promoter (ELAM), 6.6 ng of the *Renilla* luciferase reporter construct (β-actin-*Renilla* luciferase, used as a control for transfection efficiency), and varying amounts of PDisplay (an empty vector) to reach a final volume of 2 μg of DNA in each well. After 24 h of transfection, cells were then stimulated for 4 h with 10 µg/mL of purified intact LPG extract, 1 nM of PamCys3 or medium alone (control samples). Finally, transfected HEK 293 T cells were lysed and luciferase activity was assessed by NF-κB activation using the Dual-Luciferase Reporter Assay System in accordance with manufacturer instructions. Relative luminescence units (RLU) were quantitated using a SpectraMaxL luminometer. The values obtained for stimulated cells were relativized using values from non-stimulated cells. Luciferase activity is expressed as the ratio of NF-κB-dependent firefly luciferase activity to constitutively expressed *Renilla* luciferase activity. PPAR-γ expression was evaluated by western blotting after LPG stimulation in the four groups of transfected HEK 293 T cells using β-actin as a housekeeping gene.

### Statistical analysis

All assays were performed using at least three replicates per group and each experiment was performed in triplicate. Data, presented as means and SE (standard error) from representative experiments, were analyzed using GraphPad Prism 5.0 software (GraphPad Software, San Diego, CA, USA). Comparisons between groups were analyzed using the ANOVA with Student Newman-Keuls multiple comparison was used as post-hoc test. HEK 293 reporter assay where analyzed by ANOVA followed by Tukey multiple comparison post-hoc test. Differences were considered statistically significant when p ≤ 0.05.

## Electronic supplementary material

Suplemmentary Information
