# Supplementary figures and images for: Leishmania infantum lipophosphoglycan induced-Prostaglandin E2 production in association with PPAR-γ expression via activation of Toll like receptors-1 and 2
Source: Sci Rep. 2017 Oct 30;7:14321. doi: 10.1038/s41598-017-14229-8 (PMC5662570; doi:10.1038/s41598-017-14229-8)

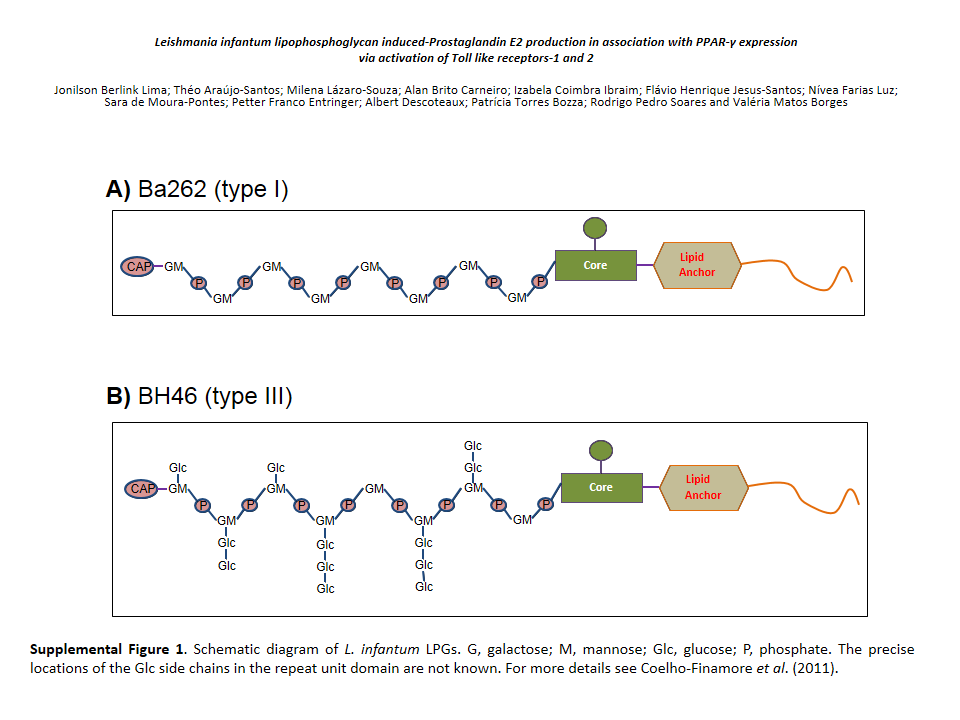

Supplement: Supplementary file 1 — Suplemmentary Information [file 41598_2017_14229_MOESM1_ESM.tif]
